# Microbiological quality assessment of fish origin food along the production chain in upper Blue Nile watershed, Ethiopia

**DOI:** 10.1002/fsn3.3147

**Published:** 2022-11-18

**Authors:** Birhan Agmas Mitiku, Marshet Adugna Mitiku, Gizachew Gelaw Ayalew, Halo Yohans Alemu, Umer Masrie Geremew, Mekidm Tamer Wubayehu

**Affiliations:** ^1^ Department of Veterinary Science, College of Agriculture and Environmental Science Bahir Dar University Bahir Dar Ethiopia; ^2^ Ethiopian Institutes of Agricultural Research National Fishery and Aquatic Life Research Center Sebeta Ethiopia; ^3^ Bahir Dar Poly Technic College Bahir Dar Ethiopia

**Keywords:** *E. coli*, fish food, microbial load, *S. aureus*, *salmonella*, *Shigella*

## Abstract

Pathogenic microorganisms can grow accidentally on fish origin human food and can be a cause of human food‐borne illness. The purpose of this study was to estimate the occurrence and microbial load pattern of *Escherichia coli*, *Salmonella*, *Staphylococcus aureus*, and *Shigella* spp. along the fish origin food value chain. A total of 396 fish samples were collected by a systematic random sampling technique of cooked and raw in the three species of fish. Fish muscles were tested using selective media, followed by conventional biochemical tests. The bacterial load was assessed using a standard plate count method. Whereas the fungal load were measured by cultured in a Sabouraud's dextrose agar (SDA) medium. The overall prevalence was *Escherichia coli* 84 (21.21%), *Salmonella* 27 (6.82%), *Staphylococcus aureus* 19 (4.80%), and *Shigella* spp. 17 (4.29%). The average mean total coliform count was observed 1.2 × 10^2^ cfu/g and 5.10 × 10^4^ cfu/g in cooked and raw fish samples, respectively. Whereas total viable count mean of 8.05 × 10^4^ cfu/g and 11.5 × 10^4^ cfu/g in cooked and raw fish, respectively. The Fungal load counts under the range 5.6 × 10^1^ cfu/g to 1.09 × 10^3^cfu/g were observed. The study has revealed that fish food in the study area has the possibility of microbial public health risk. Hence, it could be wise to improve the knowledge of key actors from harvesting to consumption to enhance the meals protection practices and high‐quality standards of fish foods.

## Graphical abstract

The microbiological contamination of fish and fish products can be a cause of human food‐borne illness. The occurrences and microbial load pattern of *Escherichia coli*, *Salmonella*, *Staphylococcus aureus*, and *Shigella* spp. and fungus in the fish origin foods along the value chain of the upper water shed of the Blue Nile River, Ethiopia, was estimated. The study has revealed that fish food in the study area has the possibility of microbial public health risk. Hence, it could be wise to improve the knowledge of key actors from harvesting to consumption to improve the food safety practices and quality standards of fish food.

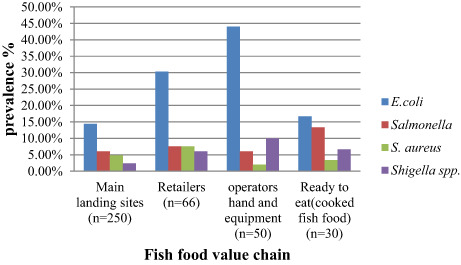

## 1 INTRODUCTION

Fish and fishery products are the most necessary nutritious meals all over the world, which represent around 15–20% of all animal protein on a world basis (FAO, 2009). Fish gives 19% of animal protein consumption in Africans and performs a special role in supplying a range of micro nutrients and especially essential fatty acids which cannot substitute food commodities. In Africa, fish consumption is 10.8 kg/person/year (Tran et al., 2019). In Ethiopia, fish consumption is 0.2 kg/person/year (Breuil & Grima, 2014).

Numerous studies have been properly demonstrated that fish origin food has essential nutrients, such as proteins, minerals, vitamins; peptides, amino acids, selenium, and long‐chain n‐3 polyunsaturated fatty acids (LC n‐3 PUFAs). In addition to nutritional value, fish food consumption has been related to protective outcomes in opposition to cardiovascular disease (CVD) and has also been related to extended fetal and child development, as well as really helpful results in various different illnesses and clinical conditions (Rimm et al., 2018). The health‐promoting effects have mainly been attributed to the LC n‐3 PUFAs, Eicosapentanoic acid (EPA), and docosahexaenoic acid (DHA) (Zenebe et al., 2006).

However, in addition to the benefits, there are risks associated with bacterial contamination and other biological, chemical, and physical contaminations. Among the risks, microbiological contamination is the leading cause of fish food (WHO, 2007). As a result, fish food is a common source of food poisoning, causing illnesses with various levels of severity ranging from mild indisposition to persistent or life‐threatening illnesses (Goja et al., 2013). Microbial contamination, in addition to its health effects, can be a cause of food wastage. Thirty percent of the fish captured are lost due to microbial activity alone (Ghaly et al., 2010).

Food‐borne diseases are recognized to regularly occur and are related to low‐income countries, probably due to improper food handling, hygiene, lack of food safety laws and weak implementation systems, lack of economic assets to procure safety tools, and lack of education and/or training for different food handlers (Addis & Sisay, 2015; Goja et al., 2013; Haileselassie et al., 2013). In Ethiopia, animal and fish origin meals are the main sources of food‐borne ailments due to poor handling conditions and sanitation practices, inadequate food safety laws, weak regulatory structures, and lack of training for food handlers (Dabassa & Bacha, 2011; Fratamico et al., 2005). This low food safety and quality practice in developing countries aggravates fish food spoilage and contamination.

Of the range of pathogenic bacterial species that cause fish food‐borne diseases, *E. coli, Sallmonella Shigella spp*., and *S. aureus* (Food and Drug Administration's [FDA's], 2021; Gosa, 2015). The occurrence of these bacteria in food is directly related to contamination. For instance, if *E. coli* count in food sample is greater than 100 cfu/g, it is unacceptable for consumption and indicates a stage of contamination (CFS (Centre for Food Safety), 2014).

Hence, monitoring of bacterial load and its occurrence pattern in fish was of supreme importance in order to provide useful data regarding the public health risk profile of fish and fish products. This work result will aid in the development of appropriate management strategies for fish food‐borne diseases. Therefore, the objective of this study was to evaluate the occurrence of bacteria (*E. coli, Salmonella, S. aureus*, and *Shigella spp*.), their load, and fungal load along the fish value chain in the upper Blue Nile watershed.

## 2 MATERIALS AND METHODS

### 2.1 Description of study area

The study was conducted in Lake Tana and surrounding districts, in the upper Blue Nile River watershed area. It is located in the northwest part of Ethiopia. The lake is one of the largest (3600 km^2^) and best fishing sites in Ethiopia. Lake Tana is found at latitude 12°1′35.75″ N and longitude 37°18′12.54″ E. Temperatures range from 13°C to 22°C on an annual basis. The annual average rainfall of Lake Tana is 1248 mm (Stave et al., 2017).

The lake has three main commercially important fish groups such as: large *Labeobarbus spp*., African catfish (*Clarias gariepinus*), and Nile tilapia (*Oreochromis niloticus*). They are consumed by a larger part of the community and traded widely in the region, even in neighboring Sudan in dry form. There are 55 fishery enterprises and a total of 21,084 beneficiaries directly dependent on the fishing activities (Mengistu et al., 2017; Shewit et al., 2018). There are 10 geopolitical districts surrounding Lake Tana with their landing sites (Dembiya, Alefa, Takusa, Northachefer, Bahir Dar Zuria, Libokemkem, Dera, Fogera, Gondar Zuria, and Bahir Dar Town).

### 2.2 Targeted population and sample types

Fish were collected at six landing sites of Lake Tana (*n* = 250), and samples were taken from randomly selected from list of local fish retailers' (*n* = 66), fish restaurants and/or hotels (*n* = 30), operator hand swab (*n* = 18), filleting knife (*n* = 16), and filleting table/board/plastic sheet (*n* = 16) of the study area. In all, 396 samples were processed. The above (*n* = 250) sample was divided according to the site and species of fish. For instance, according to the fish species, African catfish (*Clarius gariepinus*) (*n* = 60), Nile tilapia (*Oreochromis niloticus*) (*n* = 102), and *Labeobarbus spp*. (*n* = 88) were collected. Filleted raw and cooked products were chosen for sampling. A fillet includes the flesh tissue part of fish.

### 2.3 Study design, sampling and sample analysis methods

The study was carried out from October 2019 to June 2021. A total of 396 (366 raw and 30 cooked) fish samples were collected. A systematic random sampling method was employed periodically to select the sample. A quantity of a 300 g sample was taken from filleted fish muscle tissue aseptically. Each specimen was placed in a plastic bag, sealed and kept in an ice bag, and transported within 4 h until the start of sample preparation and analysis.

Sampling from the hands of fish operators, the hands' backs, palms, and fingertips were rubbed with sterile saline dipped cotton swabs. Similarly, a cotton swab sample was also collected from a knife and a filleting /cutting board/ plastic sheet. Then, the swabs were removed and placed in a triangular flask container containing 50 ml of sterile saline, which was shaken strongly to make the 1:1 sample liquid. Collected samples were tagged with identification code and transported to Bahir Dar University for laboratory processing.

Laboratory procedures were conducted using selective culture media for each tested bacteria according to the codex of bacteriological analytical methods for food samples (FAO, 2011; WHO, 2015). Following that, biochemical tests for organism isolation and identification were performed in accordance with ISO 6887‐3: (2017) recommendation for microbiological evaluation of fish samples.

The bacterial load (total viable count and total coliform count) of uncooked and cooked fish samples was assessed using the standard plate count method. The colonies were counted using a digital colony counter for each plate and expressed as colony‐forming units of the suspension (cfu/g). The fungal load was measured by culture in a Sabouraud dextrose agar (SDA) medium (Gebala & Sandle, 2013). Then, the plates were incubated at room and body temperature to check dimorphism (25°C and 37°C) for 3–7 days. Then, the results were also presented/read using cfu/g.

#### 2.3.1 Identification of *E. coli*

25 g of raw or cooked fish samples was homogenized for 2 min. in a sterile bag containing 225 ml of buffered peptone water (0.1%) (Lab M, UK) using a stomacher (Seward Stomacher 400 circulator, UK). All samples were inoculated onto eosin methylene blue medium (EMB) agar and incubated at 37°C for 24 h. Suspected colonies on EMB agar showed a green metallic sheen appearance and were subcultured on MacConkey agar medium and nutrient agar to get a pure colony and were incubated at 37°C for 24 h (Gupta et al., 2013). Colonies of *E. coli* on EMB suggest a green metallic sheen appearance. Colonies suspected to be *E. coli* were subjected to biochemical identification (Alexander et al., 2010).

The suspected result from the above media was inoculated into nutrient agar and tested by different biochemical tests: Indole test, Methyl red test, Simon citrate test, Triple sugar iron agar (TSI) test, and Urease test.

#### 2.3.2 Identification of *Salmonella*

A 25‐g portion of fish muscle tissue was placed in a sterile plastic bag with 225 ml of buffered peptone water and homogenized with a stomacher. The combined sample was homogenized and incubated for 24 h at 37°C for pre‐enrichment broth media. Thus, 1 ml of aliquot (pre‐enrichment broth) inoculated into a tube containing 10 ml of Muller Kauffmann Tetrathionate broth (MKTT broth) and a Modified Semisolid Rappaport Vassiliadis broth (MSRV broth) was inoculated with around 50 μl of broth. Both media were incubated at 37°C for 24 h.

A loop full of (approximately 10 μl) MSRV broth and MKTT broth inoculums was transferred and streaked separately onto the surface of Xylose Lysine Deoxycholate agar (XLD agar); (Titan Biotech Ltd., Bhiwadi, India) and Ramback agar plate selective media separately. The plates were incubated at 37°C for 24 h. After proper incubation, the plates were examined for the presence of suspected *Salmonella* colonies; brown, gray, or pink colonies having a black center were taken for further biochemical confirmation of *Salmonella* (Mooijman et al., 2019).

*Sallmonella* suspected colony of culture test was biochemically confirmed by inoculating into triple sugar iron agar, methyl‐red‐Voges‐Proskauer broth, indole test, Simmons' citrate agar, urea agar, lysine iron agar, kligler's iron agar, and SIM medium, then incubating at 37°C for 24 hours (Mooijman et al., 2019).The result was interpreted according to ISO‐6579‐1, (2017).

#### 2.3.3 Identification of *Shigella spp*.

25 g of homogenized sample was added into 225 ml of Gram‐negative (GN) (HuanKai Microbial, China) enrichment medium. After overnight incubation at 37°C, a loop full of the enriched culture was directly streaked onto Hektoen Enteric Agar (HEA) (HuanKai Microbial, China) and incubated aerobically at 37°C for 24 h. Then, the culture plates were examined for the presence of *Shigella* (small greenish colonies) (FDA, 2017; Trofa et al., 2009).

Colonies suspected to be *Shigella* were further characterized by standard biochemical tests; including triple sugar iron (TSI) agar, Urea Agar Base (Oxoid Ltd., Basingstoke, Hampshire, England), and the SIM test (Himedia Laboratories Pvt. Ltd. India) (H_2_S production, motility). The result of each biochemical test culture was read after incubation for 24–48 h at 37°C (FAO, 2018; FDA, 2017; WHO, 2015).

#### 2.3.4 Detection of *S. aureus*

Occurrence and identification of *S. aureus* from fish samples was made by taking 25 gram of fish muscle and transferring it aseptically into a sterile stomacher bag containing 225 ml of buffered peptone water and homogenizing it for 1–3 min using a stomacher. A 10‐fold serial dilution was prepared by transferring 1 ml of the homogenized sample to 9 ml of diluents. From appropriate serial dilutions, 0.1 ml aliquots was spread on mannitol salt agar and incubated for 24–48 h at 37°C. Presumptive colonies were inoculated on Blood Agar plates (5% difibrinated sheep blood), and the plates were incubated aerobically at 37°C and examined after 24 h of incubation for growth and hemolytic pattern of *S. aureus*. Furthermore, the colonies were identified on the basis of staining reaction with gram's stain, pigment production, colony morphology, catalase test, and coagulase test. On blood agar, *S. aureus* displays a light to golden yellow pigment and colonies surrounded by zones of clear alpha hemolysis. Inoculated media were incubated as a negative control to check for sterility.

### 2.4 Statistical analysis

Findings of this study were statistically analyzed with SPSS version 23. Descriptive statistics were made for cooked or raw fillet samples for the site; a stage of the fish value chain of total aerobic plate count and coliforms was conducted.

### 2.5 Ethical appraisal

The study protocol was revised and accepted by the research ethics appraisal board of Bahir Dar University (Ref. No. 1/3040/1.2.9). Furthermore, verbal consent was gained from all study participants aforementioned to involvement by explaining positive and negative consequences. Study units were knowledgeable that they could withdraw their involvement without restriction at any time if they felt any discomfort. Privacy of the collected information and laboratory test results were kept.

## 3 RESULTS

### 3.1 Occurrences of indicator Bacteria's

Frequency data for all bacteria studied (individually and in combination) for a total of 396 (366 raw and 30 cooked) collected over 1 year and nine‐month survey period were summarized in Table 1. The percentage (%) of positives for each disease (*E. coli, Salmonella, S. aureus*, and *Shigella spp*.) was included in the table. Overall, the results revealed that the prevalence of bacterial contamination in the fish food was variable and ranged from 0% to 44.44%. Of a total of 396 samples taken, 84 (21.21%), 27 (6.82%), 19 (4.80%), and 17 (4.29%) were positive for *E. coli, Salmonella, S. aureus, and Shigella spp*. Isolates, respectively. Of the filleted fish samples from landing sites, 18 (17.65%) of Nile Tilapia, 10 (11.36%) of Labeo Barbus, and 8 (13.33%) of African catfish were positive for *E. coli*.

**TABLE 1:** Bacterial contamination in fish origin food in our study area

Target population	Category	No of samples	No of positives (%)			
			*E. coli*	*Salmonella*	*S. aureus*	*Shigella spp*.
Species	African Cat fish (*Clarius gariepinus*)	60	8 (13.33)	3 (5)	3 (5)	1 (1.67)
	Nile tilapia (*Oreochromis niloticus*)	102	18 (17.65)	6 (5.88)	4 (3.92)	4 (3.92)
	*Labeobarbus spp*.	88	10 (11.36)	6 (6.82)	5 (5.68)	1 (1.14)
Landing sites/location	Bahir Dar	54	8 (14.81)	6 (11.11)	5 (9.26)	2 (3.70)
	Gorgora	42	5 (11.90)	1 (2.38)	1 (2.38)	1 (2.38)
	Aged kergna	42	6 (14.29)	0	0	0
	Kunzela	38	4 (10.53)	2 (5.26)	1 (2.63)	1 (2.63)
	Esay Debre	32	5 (15.63)	2 (6.25)	0	0
	Delegie	42	8 (19.05)	4 (9.52)	5 (11.90)	2 (4.76)
Fish retailer's	Whole sellers	32	9 (28.13)	2 (6.25)	2 (6.25)	2 (6.25)
	Cooperatives	34	11 (32.35)	3 (8.82)	3 (8.82)	2 (5.88)
Fish restaurants and hotels	Ready‐to‐eat cooked fish	30	5 (16.67)	4 (13.33)	1 (3.33)	2 (6.67)
Operators	Hand swab	18	8 (44.44)	2 (11.11)	1 (5.56)	3 (16.67)
	Filleting knife	16	7 (43.75)	0	0	1 (6.25)
	Filleting table/board/plastic sheet	16	7 (43.75)	1 (6.25)	0	1 (6.25)
Ground total		396	84 (21.21)	27 (6.82)	19 (4.80)	17 (4.29)

**FIGURE 1:**
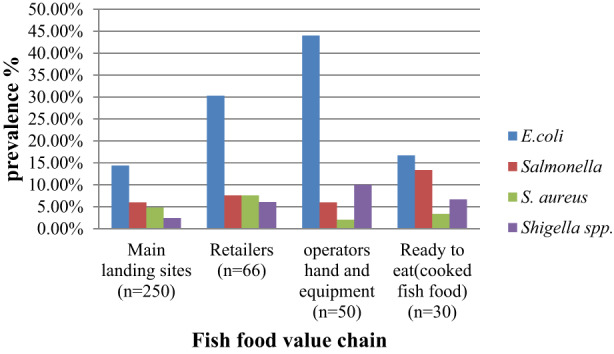
Bacterial prevalence along the fish food value chain in the study area

The prevalence of indicator bacteria's situational analysis along the fish food value chain in the study area was also documented. The first value chain identified as a source of contaminants was landing sites, retailers, operators' hands and equipment, and ready‐to‐eat (cooked fish food). The main landing sites were chosen based on the following criteria: (i) a high human population density; (ii) a high amount of fish produced and collected; (iii) the existence of regulatory bodies; (iv) the presence of fish producer cooperatives and suppliers; and (v) geographical representativeness of the lake. Specifically, the landing sites (Bahir Dar, Gorgora, Aged kergna, Kunzela, Esay Debre, Delegie) were selected. In the above‐identified fish food value chain, the prevalence of indicator bacteria was up to 44.44% *E. coli* (Figure 1).

### 3.2 Overall bacterial and fungal contamination status

Enumeration of microbial growth was carried out in all positive samples, irrespective of the pathogen. Most of the microbial load was found to be above 100 cfu/g, a level deemed to represent a risk of public health. Total coliform, an indicator of fecal contamination, was found to be an average of 5.10 × 10^4^ CFU and 11.5 × 10^4^cfu/g of total viable count in raw fish origin food samples (Table 2).

**TABLE 2:** Total bacterial and fungal load count from cooked and raw fish samples

Measure	Samples type	Minimum count	Maximum count	Average count
Total coliform	Cooked	1.4 × 10^2^ cfu/g	2.217 × 10^3^ cfu/g	1.2 × 10^2^ cfu/g
	Raw (uncooked)	1.2 × 10^2^ cfu/g	1.02 × 10^5^ cfu/g	5.10 × 10^4^ cfu/g
Total viable count	Cooked	1 × 10^3^ cfu/g	1.6 × 10^5^ cfu/g	8.05 × 10^4^ cfu/g
	Raw (uncooked)	1.92 × 10^2^ cfu/g	2.3 × 10^5^ cfu/g	11.5 × 10^4^ cfu/g
Fungi	Cooked	1.2 × 10^2^ cfu/g	1 × 10^3^ cfu/g	5.6 × 10^1^ cfu/g
	Raw (uncooked)	1.7 × 10^2^ cfu/g	2 × 10^3^ cfu/g	1.09 × 10^3^cfu/g

## 4 DISCUSSION

Despite the increasing use of fish origin food as a source of protein for nutritious meals and health benefits, there was a need to assess significant scientific evidence of microbial public health risks. Thus, this study highlights the microbial occurrences and burden of selected contamination indicator bacteria, such as *E. coli, Salmonella, S. aureus, and Shigella spp*. Due to the prevalence of bacterial contamination in the fish food in this study were 84 (21.21%), 27, (6.82%), 19 (4.80%), and 17 (4.29%) positive for *E. coli, Salmonella, S. aureus*, and *Shigella spp*. isolates, respectively.

The result of this study showed that *E. coli* prevalence was greater than the detection of Awot et al. (2019), nine (9.4%) *E. coli* isolates from 96 fish samples in fish meat retailing shops in Mekelle city, Ethiopia. But the current study finding was lower than that of Ayenadis and Aweke (2019), who reported that 80 (23.3%) *E. coli* was isolated from fish samples in Lake Hawassa, Southern Ethiopia. This result was also lower than the previous reports of Kumar et al. (2005) who estimate the prevalence of *E. coli* in tropical seafood and documented a prevalence of 47% for fecal coli forms including *E. coli*. In this study, the occurrence of *E. coli* was higher in raw fish than cooked fish samples. This might be the exposure to heat during processing the cooked fish samples. This work was agreed with the work of Gupta et al. (2013) who found 47 (48.95%) *E. coli* isolates in 96 raw fish samples and seven (12.96%) *E. coli* isolates in 88 ready‐to‐eat fish product samples. Thailambal (2006) and Omenwa et al. (2012) reported similar frequency occurrences of bacteria isolates from cooked fish foods.

Our study result was lower than Wendwesen et al. (2017), who showed 42.5% of raw fish samples had *E. coli* in Arba Minch town, Ethiopia. Moreover, this study indicated that five (16.67) of the cooked (fried fish, asa dulet, lebleb, etc…) fish samples had *E. coli*. Okonko et al. (2009) suggest that improper handling and improper hygiene might lead to the contamination of ready‐to‐eat foods, and this might eventually affect the health of consumers. The result of this study was higher than the result of Wendwesen et al. (2017) who reported that 7.5% of (asa lebleb) Nile tilapia fish muscle had *E. coli* from ready‐to‐eat fish foods in Arba Minch town, Ethiopia, and lower than Ohalete et al. (2013) report that 58.3% of fried fish had *E. coli* in Owerri, Nigeria.

With regard to the species of fish, the highest *E. coli* isolates 18(17.65%) were found in Nile Tilapia. This was agreed with previous reports by Hanson et al. (2008) who reported higher infection in plankton feeders (Nile tilapia species) of *E. coli* than in catfish. This finding disagrees with the reports of Ayenadis and Aweke (2019), who reported that there was no difference in the occurrence of *E. coli* in three species of fish. This potential disagreement might arise from the difference in sample size used, the ecosystem of the study area or sampling methods.

Of a total of 396 samples collected along the fish value chain, 27 (6.82) were found to be *Salmonella* positive (Table 2). The occurrence of *Salmonella* in this study was found to be lower than in the previous study conducted by Elhadi (2014) in Saudi Arabia, with an occurrence of 31–60% in fresh water fish samples. This result was also found to be lower than the occurrence of the previous study in China (12.4%) (Zhang et al., 2015), in Nigeria (11.5%) (Raufu et al., 2014) and higher than the study from Brazil (4%) (Ferreira et al., 2014). The reasons for these prevalence variations might be due to water quality and environmental distinctiveness (rainfall, temperature, sewage effluents; agricultural run‐off and direct fecal contamination from natural fauna) (Fernandes et al., 2018). In addition, food handlers' characteristics such as poor handling and processing practices might contribute to such a difference.

The number of *S. aureus* in the current study was 19 (4.80%). This finding was lower than with other researchers' findings in different areas of the globe. For instance, Mohammad et al. (2015) isolated 31.8% *S. aureus* in Egypt and Murugadas et al. (2016) isolated 36.5% *S. aureus* in Kerala, on the southwest coast of India. The result of this finding was also lower than that of Oh et al. (2007), Haiffa (2014), and Bujjamma and Padmavathi (2015), who reported that 17.7%, 19.8%, and 24.47% in Korea, Mosul City, Iraq, and Andhra Pradesh, India, respectively. The presence of *S. aureus* in fish indicates improper handling and possible cross‐contamination (Da Silva et al., 2020). Human beings are the major source of *S. aureus* contamination of fishery products during handling, preparation, and serving. The existence of *S. aureus* is a potential health threat to consumers, especially fish that are often eaten without further heat (Haiffa, 2014).

In the present study, a relatively lower percentage of *Shigella* spp. isolation 17 (4.29%) was recorded along the value chain of fish food samples. In fact, high levels of three (16.67%) of *Shigella* spp. were found in the hand swabs of operators. Cooking and roasting were apparently efficient in killing these pathogens (Sichewo et al., 2014). Ready‐to‐eat fish at hotels and restaurants, two (6.67%) have direct exposure to consumers. There is a small number of *Shigella* spp. that can cause infection. *Shigella* spp. organisms are highly infectious, causing bacillary dysentery after ingestion of 10–100 organisms (Kosek et al., 2010). The reason that fresh fillets had more bacteria in this study might be that they were more liable for contamination during filleting and processing.

In our study, retailers, operators' hands, and equipment were relatively the highest bacterial occurrence recorded from fish food value chain identified. The isolation of *E. coli*, *Salmonella*, *S. aureus*, and *Shigella spp*. might be attributed to sewage pollution of human origin, animal feces or unsatisfactory hygienic conditions during catching, handling, and marketing of the fish (Penakalapati et al., 2017; Food and Drug Administration's (FDA's), 2021). For instance, the fish filletors in the study area use single piece of mosquito net for decreasing the slippery of fish mucus during filleting for long period of time without disinfecting or washing. They did not wear any contamination protective wears such as gown, glove, and boots. In addition, they use single knife, without disinfecting for long period of time, plastic sheet on contaminated ground (they did not use tables), number of peoples move here and there in filleting places, and the presence of high burden of fly may increase the contamination in our study area. The most fishing style is netted overnight it is not fresh and uses contaminated nets. In all circumstances of the fish value chain, product contamination has great public health significance due to its liable nature to pathogenic bacteria from human reservoirs during handling, processing, and packaging of fish (Ghanem et al., 2019). In general, the results obtained from this study give evidence of the unsatisfactory microbiological quality and safety of fish from the local artisanal fish value chain. The particular concern was the occurrence of key indicator pathogenic bacteria such as *E. coli, Salmonella, S. aureus*, and *Shigella* spp. as fish and their products contain hazardous microorganisms. The current results of *E. coli, Salmonella, S. aureus*, and *Shigella* spp. were not compatible with the standards of the (CFS (Centre for Food Safety), 2014) and the (APHA and WHO, 2004) and thus pose a significant public health risk.

This study's results indicate that a low mean value of bacterial load was found in ready‐to‐eat or cooked fish samples. This might be due to the negative effect of heat on the bacteria during fish origin food preparation. Our finding was that there was a lower microbial load compared with the results of Wendwesen et al. (2017) who reported that there were 4.63 × 10^6^ cfu/g in frozen raw Nile Tilapia fish samples and 4.92 × 10^3^ cfu/g in inadequately cooked fish origin foods in Arba Minch town, Ethiopia. The coliform load in raw fish samples was higher than the result of Dhanapal et al. (2012), who found 4.9 × 10^4^cfu/g and lower than the result of Wendwesen et al. (2017), who reported 4.63 × 10^6^cfu/g in frozen raw Nile tilapia fillet samples. The mean fungal load was measured by culture in a Sabouraud dextrose agar (SDA) medium and was 1.09 × 10^3^ cfu/g and 5.6 × 10^1^ cfu/g in raw and cooked fish samples, respectively. This non‐negligible high microbial load in our study area might be due to the result of poor handling during transportation and/or poor personal hygiene during harvesting and filleting.

The Center for Food Safety organization has set minimum standards for the recovery of microorganisms from foods of various origins. When compared with that standard, the recovery rate in the current study result was higher, and this could be due to the absence of hygienic practices and strict follow‐up of this sector by the concerned authorities. According to CFS (Centre for Food Safety) (2014) guidelines, <20 cfu/g is satisfactory, 20–10^2^ intermediate or border line and >10^2^ unacceptable for human consumption. The average microbial load (total viable count and coliform count) of the fish samples was in an unacceptable range.

## 5 CONCLUSIONS

Most of the fish samples were found to contain higher microbial loads than prescribed standards. This indicates *that E. coli, S. aureus, Salmonella*, and *Shigella* spp are contaminants of fish in the study area, and their occurrence in fish could represent a risk to consumers. The total coliform, total viable, and fungal/mold count indicate that they were above the recommended level of the Centre for food safety standards. Due to this, fish handlers and processors should be trained on the hygienically and sanitary handling of foods. The sanitation of the filleting ground, fish harvesting materials, and fish landings in the study area should be kept clean. This indicates that there is an inadequate level of food safety and poor handling practices at every stage of the fish value chain. Therefore, fish harvester societies and handlers should undertake training and education on regarding safe food handling to prevent contamination. Processing plants should be built at each landing site with their facilities. A new code of practice to control infections of fish origin food should be installed in study area to ensure safe fish products. Education and training should be given to fish handlers.

### 5.1 Limitations

Confirmation of bacteria by molecular methods was not performed. Similarly, serotyping of isolated pathogens was not done.

## FUNDING INFORMATION

Ethiopian Institute of Agricultural Research; National Fishery and Aquatic Life Research Centre; ref. No. 17.2/0409/2019.

## CONFLICT OF INTEREST

The authors declare that they have no competing interests.

## Data Availability

All data and materials are available upon reasonable request from the corresponding author.
